# Heat stress and amphibian immunity in a time of climate change

**DOI:** 10.1098/rstb.2022.0132

**Published:** 2023-07-31

**Authors:** Louise A. Rollins-Smith, Emily H. Le Sage

**Affiliations:** Department of Pathology, Microbiology and Immunology, Vanderbilt University School of Medicine, Nashville, TN 37232, USA

**Keywords:** amphibians, desiccation stress, heat stress, HPI axis, immune defences, microbiome

## Abstract

As a class of vertebrates, amphibians, are at greater risk for declines or extinctions than any other vertebrate group, including birds and mammals. There are many threats, including habitat destruction, invasive species, overuse by humans, toxic chemicals and emerging diseases. Climate change which brings unpredictable temperature changes and rainfall constitutes an additional threat. Survival of amphibians depends on immune defences functioning well under these combined threats. Here, we review the current state of knowledge of how amphibians respond to some natural stressors, including heat and desiccation stress, and the limited studies of the immune defences under these stressful conditions. In general, the current studies suggest that desiccation and heat stress can activate the hypothalamus pituitary–interrenal axis, with possible suppression of some innate and lymphocyte-mediated responses. Elevated temperatures can alter microbial communities in amphibian skin and gut, resulting in possible dysbiosis that fosters reduced resistance to pathogens.

This article is part of the theme issue ‘Amphibian immunity: stress, disease and ecoimmunology’.

## 1. Amphibians responding to changing environments

Amphibians are ancient creatures valued by all human societies. They play critical roles in aquatic and semiaquatic environments as important consumers or competitors of insects and as prey for other animals. They share a complex neuroendocrine system with other vertebrate species that enables them to thrive in a variety of environments (reviewed in [1]). Given their long evolutionary history, it is likely that some species are adapting to current climate changes, but there is a concern that some are unable to adapt quickly enough, leading to losses of biodiversity.

The most recent technical report of the Intergovernmental Panel on Climate Change (IPCC, the United Nations body for assessing the science related to climate change) indicates with high confidence or very high confidence that species in all ecosystems have begun to shift their geographical ranges and alter the timing of seasonal events in response to a warming climate (https://www.ipcc.ch/report/sixth-assessment-report-working-group-ii/) [2]. Many species of amphibians have wet skin with higher evaporative water loss than reptilian and mammalian skin and use the skin for both respiration and regulation of essential ion balance (reviewed in [3]). Thus, they are likely to be among the species most affected by climate change, with expectations that ranges for some species will contract until no suitable habitat will remain, especially in tropical regions and the Amazon [4,5]. Using more than a decade of observations, Muths *et al*. [6] demonstrated that for temperate amphibian species, population dynamics were influenced by climate change, though responses were highly variable and context-dependent. In temperate regions of the midwestern USA (Minnesota and Wisconsin), evidence suggests that calling and breeding started earlier in some warm years compared with historical records dating back to 1895 [7], and some western habitats are becoming warmer and drier [8]. For example, Yellowstone National Park in the western USA has warmed significantly since 1980 [9]. Effects of warming include lower snowfall at high elevations, which leads to shorter amphibian habitat persistence, lower breeding success [10] and lower overwintering survival, especially of toads infected with the chytrid fungus *Batrachochytrium dendrobatidis* [11]. Soils there and elsewhere in the western USA have become drier, and these trends are expected to continue [12]. In a study of Florida, USA wetlands, Greenberg *et al*. [13] used 17 years of temperature, rainfall and water depth measurements to develop a model to forecast water depths of ephemeral wetlands out to the year 2060. Their prediction was that only one of five amphibian species that are currently present would thrive under these conditions. Thus, it seems that, to survive, populations will need to continue to shift their ranges and evolve greater tolerance to warmer, drier conditions. Such range shifts may incur costs in terms of immune defences. Two studies of invasive amphibian species (Cuban treefrogs, *Osteopilus septentrionalis*, and cane toads, *Rhinella marina*) expanding their range in the state of Florida, USA showed that those toads at the leading edge showed diminished activity of one key measure of innate immunity in their plasma. Bacterial killing activity (BKA) was decreased, suggesting poorer complement activity [14,15]. Invasive cane toads at the expanding edge of their range in Australia also showed somewhat poorer responses in measures of a cell-mediated lymphocyte response. Toads from older-established populations away from the invasion front had more circulating white blood cells and recruited more white blood cells into toe-webbing following injection with a plant lectin, phytohaemagglutinin (PHA), than newer populations of toads at the expanding invasion front [16]. Although the authors did not measure corticosterone in the Cuban treefrog study, they suggested in the discussion that toads at the leading edge may have had elevated corticosterone because of increased metabolic needs associated with movement. This might have explained the reduced immune functions. In another study using invasive cane toads expanding their territories in Florida, this hypothesis did not seem to be supported. That is, baseline levels of corticosterone were not different between the northernmost populations at the leading edge. The invasive cane toads at the northern edge of their habitat range expansion in Florida had a poorer corticosterone response to short-term stress whereas the warmer, more established southern populations responded better with elevated corticosterone responses [15]. All of these studies suggest possible trade-offs between the need to support metabolism in marginal habitats and to support of immune defences.

Many amphibian species depend on precipitation-fed freshwater habitats [17], which are experiencing greater frequency and severity of droughts with climate change [18]. Given the persistence of water in these habitats can be variable year to year, some amphibians have remarkable adaptations to sense declining water levels to accelerate larval development and escape the drying pond (reviewed in [19,20]). Although plasticity can increase the chances of surviving in variable environments, exposure to pond drying typically results in faster development at a cost of a smaller size at metamorphosis, leading to lower survival and fecundity (reviewed in [21]). Because the hypothalamus–pituitary–interrenal (HPI) axis orchestrates both the accelerated metamorphosis phenotype [22] and the drastic immune system changes that occur with metamorphosis [23], researchers have hypothesized immune trade-offs are likely to occur (reviewed in [24]). Only a small number of amphibian species have been studied under shortened hydroperiod conditions (reviewed in [25,26]), and only a few have assessed immune responses. Specifically, shorter hydroperiods led to weaker cellular immune system responses to PHA in wood frogs (*Rana sylvatica*) and northern leopard frogs (*Rana pipiens*) [27,28]. On the other hand, the New Mexico spadefoot toad (*Spea multiplicata*) did not display carry-over effects of pond drying on immune function [29]. In two species of leopard frogs (*R. pipiens* and *Rana sphenocephala*), carry-over effects of shorter hydroperiods also included changes in host-associated microbiota [30], shifting to lower capacities to inhibit pathogen growth [31]. Thus, the effects of pond drying on the development of specific immune defences in postmetamorphic amphibians have been studied in a limited number of species, and further studies are needed.

## 2. Effects of heat and dehydration as stressors on immunity in adult amphibians

In this section we examine what is known about the effects of extreme heat and/or dehydration on the ability of adult amphibians to mount effective immune responses. It should be noted that amphibians are a very diverse class of animals with variable thermal tolerance limits [32], and the effects of extreme heat and desiccation on immune function have not been well studied. Most studies have been conducted with anuran species, and there are very few studies on urodeles or caecilians. Thermal performance can also vary by populations within a species [33,34]. Species differ in their capacity to resist evaporative water loss, and hylid frogs with higher desiccation resistance were predicted to be able to tolerate a higher range of temperatures [35]. As further evidence that species differ greatly in their responses to desiccation, a study of five species of Brazilian toads from differing habitats showed that larger species had higher rates of water uptake but lower resistance to water loss [36]. Furthermore, thermal performance curves (i.e. performance peaks at some ‘optimal' temperature and mortality occurs at upper and lower limits) (reviewed in [37]) vary by age and life stage [38]. Lertzman-Lepofsky *et al*. [39] emphasized the importance of considering both elevated temperature stress and evaporative water loss as risks for reaching the physiological limits of amphibians as the Earth warms. For example, using biophysical models based on empirical hydrothermal performance curves, Greenberg & Palen [40] demonstrated that both thermal and hydration physiology need to be considered when estimating climate change effects on amphibians. Behavioural changes that allow amphibians to move to a warmer temperature setting have the potential for the amphibians to avoid chytridiomycosis caused by the chytrid fungi *B. dendrobatidis* and *Batrachochytrium salamandrivorans* [41,42]. However, in a natural setting in Belgium, salamanders in the field rarely achieved the temperature needed to resist infection by *B. salamandrivorans* [42]. Thus, the need of some amphibians to remain in cool wet environments precluded their ability to avoid disease.

On a positive note, some recent publications suggest that amphibians have both behavioural and physiological plasticity that may enable them to adapt and evolve to changing thermal conditions ([43], reviewed in [44]). An example may be found in plethodontid salamanders in the Appalachian Mountains of the USA. A recent study showed that six of fifteen species studied showed significant reductions in body size over the last 55 years as a response to increasing temperatures, especially at southern latitudes with hotter drier conditions. Possible mechanisms for the reduced body size include reduced foraging success under suboptimal conditions resulting in reduced overall growth [45]. Another example of decreased body sizes over many decades was found in a study of frogs in museum collections from Borneo that were linked with climate records spanning more than 100 years. One conclusion of this study was that frogs were larger under wet conditions than in dry conditions at cool temperatures, suggesting that when resources were limited at colder temperatures, body size was reduced [46].

### (a) Effects of desiccation independent of heat stress on immunity in adult amphibians

Free-living amphibians experience many weather-related changes throughout their lives, and the release of glucocorticoid hormones owing to activation of the HPI axis is thought to be important for energy balance during stressful and non-stressful conditions [47]. The main glucocorticoid hormone in amphibians is corticosterone, and the main mineralocorticoid is aldosterone. Both hormones are involved in normal development, energy mobilization, and osmoregulation, and both can inhibit lymphocyte proliferation and induce apoptosis of lymphocytes in tadpoles and adult frogs (reviewed in [1]).

There are limited studies of the effects of desiccation alone on amphibians, but many studies have documented that adaptations used to survive dehydration can be costly in terms of energy (i.e. increased heart rate and cardiac contractility), taxing on cardiovascular tissues (i.e. increased blood hyperosmolality, hypovolaemia and hyperviscosity), and cause the release of reactive oxygen species (reviewed in [48]). In terms of immune function under desiccation conditions, several studies of invasive species at invasion fronts in arid climates have demonstrated changes in immune functions of the dispersing populations. The expanding populations of the guttural toad, *Sclerophrys gutturalis*, in South Africa showed poorer hydration and an apparent higher BKA under field conditions [49]. However, in ornate forest toads (*Rhinella ornata*), natural desiccation resulted in elevated corticosterone (81 and 282 ng ml^−1^ when dehydrated by 10 and 20%, respectively). Under these stressful conditions, the numbers of circulating lymphocytes were reduced, while the numbers of circulating neutrophils were increased, suggesting a possible effect of the stressful conditions on immune parameters [50]. In crab-eating frogs (*Fejervarya cancrivora*), which inhabit mangrove swamps and marshes in Southeast Asia, dehydration increased both aldosterone and corticosterone levels (approx. 20–30 pmol ml^−1^ aldosterone, approx. 50–85 pmol ml^−1^ corticosterone) [51]. Dehydration increased aldosterone in cane toads (*R. marina*) dehydrated by lack of access to water (40 pmol ml^−1^ in plasma) [52]. Likely these documented elevations in osmoregulatory hormone levels (the mineralocorticoids aldosterone and corticosterone) are protective during periods of dehydration, but whether these hormonal changes are immunomodulatory depends on their duration and magnitude (reviewed in [53]).

### (b) Effects of heat stress on immunity in adult amphibians

As ectotherms, amphibian metabolism increases with temperature [54], resulting in greater energetic demands which could exceed available resources. Amphibians can respond to extreme heat through behavioural changes such as seeking cooler areas underground or underwater. Hypothetically, if high metabolic costs are accrued at upper thermal limits, less energy may be available to mount effective immune responses, although, physiological trade-offs may prioritize immune function in certain contexts. Further, various immune functions likely have distinct but related thermal performance curves [55,56]. Hotter conditions can also trigger physiological changes mediated by the HPI axis, given that glucocorticoids and metabolism are generally thought to positively covary (reviewed in [57]). For example, exogenous glucocorticoids increased metabolic rates in one study of red legged salamanders (*Plethodon shermani*) [58]. An example of a species that has a relatively high critical thermal maximum (CT_max_; [32]) is the invasive cane toad (*R. marina*) in Australia. At the extreme end of their range in the Northwest Territory of Australia, cane toads showed increased corticosterone in blood and urine under conditions of heat stress [59–62]. A study of cane toads in a setting in which the temperature was naturally increasing during the day to very high temperatures (shade temperatures exceeded 40°C), corticosterone levels were highest at the time of day when the temperature was the greatest. Increased glucocorticoids were followed by increased evaporative water loss, suggesting cooling due to water loss across the skin. Evaporative water loss is linked to elevated temperatures as a means to cool the skin (reviewed in [63]). Daily elevated corticosterone levels for the cane toads reached on average nearly 120 ng ml^−1^. When the temperatures dropped daily, the toads moved to water sources and became hydrated. If prevented from reaching water sources, the toads died [59]. Thus, elevated glucocorticoids in this setting appear to be an adaptive response resulting in increased evaporative water loss cooling the toads, and the water loss would be replaced to permit survival under these very harsh conditions. However, these levels of corticosterone would seem to be incompatible with lymphocyte viability [64,65]. Glucocorticoids likely play a role in water-seeking behaviour, as seen in guttural toads (*S. gutturalis)* when corticosterone levels were artificially increased [66]. This may be an example of a trade-off between temporary depression of immune responsiveness to permit survival.

The microbial communities of the skin and gut of amphibians are also critical for their health and survival (reviewed in [67,68]). Depletion of skin microbes by antibiotic treatment can increase pathogen susceptibility [69,70]. Thus, when thinking about the effects of heat on immunity, it is important to consider the effects of temperature changes on the microbiome. Two studies of red-backed salamanders (*Plethodon cinereus*) suggest that elevated temperatures (20–21°C, within survival range) altered the microbial communities of the skin and the gut [71,72]. For the gut microbes, the elevated temperature reduced the microbial diversity, leading to reduced capacity to digest food and an increase in a potentially pathogenic bacterial group [71]. Elevated temperature also reduced the diversity of the microbial skin community, and the diversity was further reduced when the animals were exposed to the pathogenic chytrid fungus *B. dendrobatidis* [72]. Because the soil environment is a natural source of amphibian microbial communities from which the host likely selects a subset [73,74], changes in soil temperatures would also impact availability of protective commensal microbes. Thus, temperature shifts toward the warmer end of the environmental temperature tolerance may adversely affect the microbial skin communities and resistance to disease.

## 3. Effects of natural stressors on tadpoles

Free-living tadpoles experience conditions determined by the aquatic environment in which they are hatched from eggs. They have no escape until metamorphosis, and thus at this life stage, they must also adapt by either behavioural or physiological mechanisms. However, their endocrine systems and immunological systems are still developing (reviewed in [23]). Here we discuss some examples of natural stressors and the effects on tadpole immune responses.

### (a) Effects of oxygen or food deprivation on aquatic larval stages

Aquatic larval stages could be exposed to lower levels of dissolved oxygen and food when temperatures increase. Both limited oxygen in the water and limited food sources may compromise tadpole investment in immunity. Some, but not all, species are capable of increasing oxygen uptake through aerial respiration or gulping air at the water surface [75]. This response can come at a cost of energy expenditure to swim to the surface as well as increased predation risk. There are many examples in the literature demonstrating the immunosuppressive effects of oxygen deprivation in fish [76], though we could find none on larval amphibian immunity. One study simulated future climate conditions for developing *Polypedates cruciger* (common hourglass frog) tadpoles through increasing CO_2_ and subsequently decreasing pH, which resulted in lower white blood cell counts in circulation (relative to red blood cells). However, oxygen levels were not measured in this study [77]. While amphibian larvae may have higher tolerance to hypoxia than fish, given the potential for warming and excess nutrients to increase hypoxia risk in certain ecosystems, more research is warranted here.

In a study of short-term food deprivation on western spadefoot toads (*Spea hammondii*), Crespi & Denver [78] showed that food deprivation of premetamorphic (Gosner stage 31) or prometamorphic (Gosner stage 36) tadpoles resulted in elevated corticosterone to a whole body level that would be incompatible with circulating lymphocytes [64] whereas postmetamorphic juveniles (nine months postmetamorphosis) decreased the release of corticosterone following food deprivation in favour of reduced activity. Loss of lymphocytes in tadpoles would be replaced when food resources return because the lymphocyte populations are expanding in waves during larval development [79,80]. The decreased activity in postmetamorphic toads was thought to be a strategy to conserve energy until food would become available again, but a secondary benefit, is that corticosterone was not elevated, and lymphocyte activity would not be affected in the juvenile frogs at a time when lymphocyte populations are rapidly expanding [79–82].

### (b) Effects of heat stress on aquatic larval stages

In general, there are very few published studies of the effects of heat stress on immune defences of tadpoles. However, some recent studies have examined the effects of elevated temperatures on immune cells in the blood of metamorphosing tadpoles and on the larval microbiomes.

In addition to the study of the effects of elevated CO_2_ that affects pH and white blood cell counts cited above [77], the authors examined the effects of temperatures elevated by 3 or 5°C (from 29 to 32 or 34°C) on survival and blood cell numbers in developing *P. cruciger* (common hourglass frog) tadpoles at the conclusion of metamorphosis. Both elevated temperatures reduced survival, and all the tadpoles at 34°C died before reaching metamorphosis. The tadpoles at 32°C experienced high mortality after metamorphosis in comparison with controls. At the conclusion of metamorphosis, the tadpoles at 32°C also had reduced numbers of total white blood cells relative to red blood cells in comparison with control frogs, and the proportions of lymphocytes, monocytes and neutrophils among the white blood cells were increased in comparison with control frogs. This study suggests that elevated temperatures in this tropical frog added additional stress to the haemopoietic cell compartment during the critical period of metamorphosis when lymphocyte numbers in the thymus and spleen are reduced by the glucocorticoid and thyroid hormone-driven events of metamorphosis (reviewed in [23]). This reduction in immune cells likely made the newly metamorphosed froglets highly vulnerable to infection, and in this study, many died immediately after metamorphosis.

The microbial communities that inhabit the gut and skin of larval amphibians are different from those of adults of the same species [83,84]. Elevated temperatures can alter those communities at the larval stages. For example, leopard frog tadpoles (*R. pipiens*) raised at an elevated temperature of 28°C (slightly higher than the medium preferred temperature of 20–25°C) [85] had strikingly different microbial communities from those raised at 18°C, and the warm tadpoles had a greater abundance of members of the potentially pathogenic genus *Mycobacterium* ([86], reviewed in [87]). A shift in the gut microbiome was also detected within a very short time (1–4 days) in tadpoles of green frogs (*Rana clamitans*) and American bullfrogs (*Rana catesbeiana*) when the acclimation temperature of 24°C was shifted to 29°C [88]. Additional studies of green frog tadpoles showed that reduction of the gut microbiome by rearing in sterile water reduced the thermal tolerance and survival of microbe-depleted tadpoles in comparison with tadpoles raised in water containing microorganisms [89]. Another more natural study of the gut microbiota of tadpoles of a bromeliad plant-specialist frog species found in Brazil (*Ololygon perpusilla*) showed that elevated temperatures (about 6°C above ambient) led to significant changes in the gut microbiome that were characterized as dysbiosis. The tadpoles were in competition with other invertebrates, including mosquito larvae. The warming temperatures altered the environmental bacterial community and the arthropod community such that bacterial communities in the tadpole gut changed, resulting in stunted tadpole growth [90]. All of these studies show that temperature can have a dramatic effect on the gut microbial community necessary for food digestion, adaptation to temperature changes, and survival.

Amphibian larvae are susceptible to infection by trematodes (mainly *Ribeiroia* and *Echinostoma* genera) and ranaviruses (family Iridoviridae). For example, the Pacific treefrog (*Pseudacris regilla*) is infected by cercariae of the trematode *Ribeiroia ondatrae* shed by the intermediate-host snail. Several studies suggest that warmer temperatures have differing effects on the parasite and host. A warm temperature of 26°C resulted in fewer cercariae surviving after being shed by the snail and fewer encysted parasites in the tadpoles, but this temperature accelerated development of the tadpoles. The authors attributed this greater resistance of the frog host to a possible shortening of susceptible larval stages or to enhanced immunity, including development of more eosinophils and lymphocytes [91]. The temperature of 26°C is well within the temperature tolerance of the host tadpoles [92] but had adverse effects on snail survival [93]. While immunity was not measured, larvae of several species are more likely to die from ranavirus infection when exposed at warmer temperatures, though studies found considerable interspecific variation [94,95]. More research is needed to assess how antiviral responses vary with temperature to explain differences among species.

## 4. Concluding remarks

Amphibian species at all life stages continue to be vulnerable to population declines owing to multiple interacting factors such as habitat loss, disease, environmental chemicals, invasive species, overuse by humans, and emerging diseases (reviewed in [96]). Among the newest threats is global climate change. Unpredictable temperatures and rainfall resulting from climate change will exacerbate the effects of the other factors. It is likely that some species will find ways to adapt and evolve, but other species in specialized niches or with small reproductive capacity may not adapt quickly enough. Studies of the effects of climate change on the immune system of amphibians are very limited, but they suggest that the stresses of extreme heat and drought will impact the vulnerability of amphibians to diseases such as chytridiomycosis and ranavirus outbreaks. One area that would benefit from additional research mentioned in this review is the need to understand the effects of shorter hydroperiods on developing amphibians and resulting effects on development of immunity. Another is the effect of reduced oxygen levels in aquatic environments on tadpoles and adults, which need to expend more energy to survive, and the effects of hypoxia on immunity. Most of the studies we have been able to access were from the USA or North America. However, effects of climate change on amphibians in tropical areas will likely be different, and more studies are needed from these vital tropical habitats. It is also the case that most studies of heat stress on immunity in amphibians have been conducted with anuran species (frogs and toads). More studies should also be conducted on urodeles, which seem to be especially vulnerable to the chytrid pathogen *B. salamandrivorans* [42,97,98]. Not only do we need to better understand the effects of changing environments and a changing climate on amphibian immunity, but also future research should focus on ways to mitigate climate change impacts and prioritize vulnerable species.

## Data Availability

This article has no additional data.
